# MTERFD1 promotes cell growth and irradiation resistance in colorectal cancer by upregulating interleukin-6 and interleukin-11

**DOI:** 10.7150/ijbs.36916

**Published:** 2019-10-21

**Authors:** Xiaoshuang Liu, Xiaopeng Cao, Cong Liu, Yi Cao, Quanquan Zhao, Xiaojie Tan, Xu Li, Xiaodong Xu, Enda Yu, Hao Wang

**Affiliations:** 1 Department of Colorectal Surgery, Changhai Hospital, The Second Military Medical University, Shanghai, 200433, China.; 2 Department of Gastroenterology and Hepatology, The First Affiliated Hospital of Chinese PLA General Hospital, Beijing, 100048, China.; 3 Department of Radiation Medicine, Faculty of Naval Medicine, The Second Military Medical University, Xiangyin Road, Shanghai, 200433, China.; 4 Department of Epidemiology, Faculty of Naval Medicine, The Second Military Medical University, Xiangyin Road, Shanghai, 200433, China.

**Keywords:** MTERFD1, colorectal cancer, irradiation resistance, IL-6, IL-11

## Abstract

The role of the novel oncogene, mitochondrial transcription termination factor (MTERFD1), in human colorectal cancer (CRC) is unclear. Here, we report the role MTERFD1 in CRC. We conducted plasmid construction and transfection analyses, cell proliferation assays, apoptosis detection assays, ELISA, western blotting, and qRT-PCR using cell culture applications. MTERFD1 was upregulated in human and chemically induced mouse CRC tissues. *In vitro* functional assays showed that MTERFD1 overexpression promoted human CRC cell proliferation, whereas knockdown of endogenous MTERFD1 significantly enhanced apoptosis in these cells. MTERFD1 expression was positively linked to irradiation resistance in CRC cells. Furthermore, interleukin (IL)-6 and IL-11 were identified as the effector molecules of MTERFD1 in its oncogenic role and irradiation resistance in CRC cells. Our results demonstrated that MTERFD1 played an oncogenic role in CRC development and enhanced irradiation resistance by regulating IL-6 and IL-11 in CRC cells. MTERFD1 may serve as a potential prognostic and therapeutic marker for radiotherapy in CRC.

## Introduction

Colorectal cancer (CRC) is a major contributor to cancer-related mortality and morbidity among humans worldwide. CRC is among the 5 most prevalent cancers in men and women in China and the U.S.^1^, ^2^. Radiotherapy is one of the primary comprehensive treatment methods for CRC. Response to radiotherapy varies greatly among individuals with CRC. The molecules correlated with CRC radiosensitivity must be identified to screen out radiosensitive CRC patients and assess radiation therapy prognoses. Determining the mechanism or signaling pathways of the molecules underlying radioresistance may facilitate the development of agents that can sensitize CRC to irradiation. Although some potential predictive molecules of radiotherapy sensitivity in CRC have been reported, such as p53, ATM, EGFR, K-RAS and survivin^3^-^6^, none are currently applied in clinical practice.

Mitochondrial transcription termination factor (MTERF) family proteins are reported to be primarily associated with regulating mitochondrial DNA (mtDNA) expression^7^. MTERFD1, also known as mitochondrial transcription termination factor 3 (MTERF3), is an essential gene for mouse embryonic survival. MTERFD1 inhibits mtDNA transcription initiation^8^, and MTERFD1 knockdown and knockout causes massive activation of mtDNA transcription, inhibits the large mitochondrial ribosomal subunit, and impairs ribosomal assembly, indicating its role in the crosstalk between mitochondrial transcription initiation and ribosome biogenesis^9^. In our recent work, MTERFD1 functioned as an oncogene in multiple cancers. Gene amplification and upregulation of MTERFD1 were negatively correlated with overall patient survival. MTERFD1 overexpression enhanced tumor cell growth *in vivo* and *in vitro* 10. However, the oncogenic effect of MTERFD1 in CRC remains uncertain, and the underlying molecular mechanism of MTERFD1 remains unclear.

In this study, we demonstrated that MTERFD1 promoted CRC development by regulating interleukin (IL)-6 and IL-11 in CRC cells. In addition, MTERFD1 enhanced the irradiation resistance of CRC and could serve as a potential prognostic and therapeutic marker for radiotherapy of CRC.

## Materials and Methods

### Analysis of MTERFD1 gene expression

Gene expression data were downloaded from the NCBI Gene Expression Omnibus (GEO) database (accession numbers: GSE32323, GSE8671, GSE31106). The expressions of different CRC stages were downloaded from the UALCAN online database (http://ualcan.path.uab.edu/index.html) 11.

### Cell culture and transfection

Human colorectal cancer cell lines (HCT116, SW1116, SW620, SW480, HT29, and LOVO) and human normal intestinal cell lines (NCM460 and HIEC) were obtained from the Shanghai Institute of Biochemistry and Cell Biology, Chinese Academy of Sciences (Shanghai, China). Cells were cultured in Dulbecco's modified Eagle's medium (DMEM) supplemented with 10% fetal bovine serum, 50 U/mL penicillin and 0.1 mg/mL streptomycin. All cells were maintained at 37°C in a 5% CO2 humidified incubator. Cells were seeded on the plate overnight, then transfected with 2 µg of plasmid or 50 pmol of small interfering RNA (siRNA) per transfection using Lipofectamine 3000 per the manufacturer's protocol (Invitrogen, Carlsbad, CA, USA). Cells were harvested at the indicated hours post-transfection for further analysis.

### Plasmid and siRNA

The MTERFD1 overexpression (MTERFD1-OE) plasmid (pcDNA3.1-MTERFD1) was designed and constructed as described previously 10. The blank plasmid (pcDNA3.1) was used as a control for cell transfection. Two siRNAs for MTERFD1 knockdown (MTERFD1-KD; siRNA 1: sense 5'-GACACUUGUUUCUUACCUA-3', siRNA2: sense 5'-GGCUAUUCAGAUUAUUGCA-3') and a control siRNA (sense 5'-UUCUCCGAACGUGUCACGU-3') were designed and synthesized by GenePharma Company.

### RNA extraction, reverse transcription and quantitative real-time PCR (qRT-PCR)

Total RNA was extracted with TRIzol reagent (Invitrogen, Carlsbad, CA, USA) per the manufacturer's protocol. The cDNA was synthesized by reverse transcribing the total RNA using the PrimeScript^TM^ RT reagent kit with gDNA Eraser (TaKaRa, Shiga, Japan) as per the product manual. qRT-PCR was performed using the SYBR Premix Ex Taq^TM^ II PCR Kit (TaKaRa, Shiga, Japan) and the standard protocol of the Applied Biosystem 7500 Fast Real-Time PCR System (ABI, Foster City, CA, USA). The primers used in the qRT-PCR were GAPDH (5'-CCATGTTCGTCATGGGTGTGAACCA-3' and 5'-GCCAGTAGAGGCAGGGATGATGTTC-3') and MTERFD1 (5'- AGGCTGCTAACTGGAAGTCTGG-3' and 5'-ATGATGTGGTGGGGAATGCTCA-3'). Relative mRNA levels of MTERFD1 were normalized to the GAPDH reference gene expression and calculated via the 2^-△△CT^ method.

### Western blotting

Colorectal cancer cells were trypsinized, washed twice with cold phosphate-buffered saline (PBS), and resuspended in lysis buffer (10 mM HEPES at pH 7.4, 2 mM EGTA, 0.5% Nonidet P-40, and 1 mM phenylmethylsulfonyl fluoride with 1X protease inhibitor cocktail [Sigma-Aldrich, St. Louis, MO, USA]) on ice for 30 min to extract total cell proteins. Total proteins were fractionated using 12% SDS-PAGE gel and transferred to nitrocellulose membranes. Membranes were probed with anti-MTERFD1 rabbit antibody (Sigma-Aldrich, St. Louis, MO, USA) diluted at 1:1,000 and developed with horseradish peroxidase (HRP)-conjugated anti-rabbit IgG antibody (Sigma-Aldrich, St. Louis, MO, USA) diluted at 1:5,000 and ECL Prime western blotting detection reagent (GE Healthcare, USA).

### Cell viability assay

The MTT assay (Promega, Fitchburg, WI, USA) was used to assess cell proliferation and survival per the manufacturer's instructions. Five thousand cells per well were seeded in triplicate in a 96-well plate 12 h post-transfection. Cell viability was determined by measuring the absorbance of each sample at 570 nm using a microplate reader (Infinite F50, TECN, China) every 24 hr for 3 days.

### Apoptosis detection assay

Cells under the various treatments were stained with Annexin V-FITC and propidium iodide (PI; BIPEC) per the manufacturer's instructions. Cells in each sample were tested by flow cytometer (Beckman Coulter, Indianapolis, IN, USA), and data were analyzed using CyCotExpert software (Beckman Coulter) as described previously 12, 13. Annexin V-positive cells were counted as apoptotic cells.

### Enzyme-linked immunosorbent assay (ELISA) analysis of IL-6 and IL-11 levels

IL-6 and IL-11 levels in the cell culture supernatants were analyzed using the human IL-6 and IL-11 Quantikine ELISA Kit (JIANGLAI Company, Shanghai, China) per the manufacturer's instructions. The treated cells were cultured in 6-well plates for 24-48 hours. The culture medium was collected and centrifuged at 3000 g to remove cell debris, then tested.

### Irradiation

Treated cells were irradiated with the indicated doses by ^60^Co-gamma rays at the radiation center of the Faculty of Naval Medicine, Second Military Medical University, China 12, 13.

### Statistical Analysis

The experimental groups and relevant controls were compared via Student's t-test using SPSS software. P<0.05 was considered statistically significant.

## Results

### MTERFD1 was upregulated in colorectal cancer tissues and cell lines in vivo and in vitro

**Figure 1:**
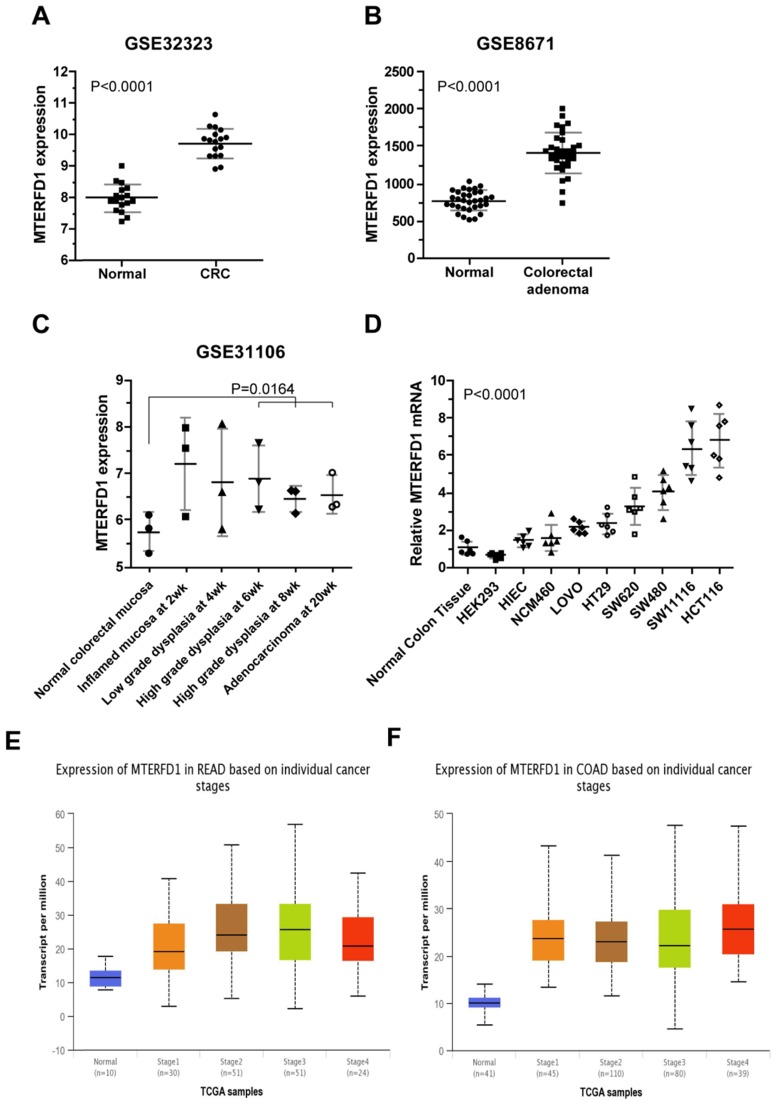
** MTERFD1 expression in colorectal cancer.** (A, B) MTERFD1 expression was increased in colorectal cancer tissues compared with adjacent or paired normal tissues in two GEO datasets. (C) MTERFD1 expression levels were compared time-dependently in mice with AOM/DSS-induced colitis-associated CRC. (D) The relative MTERFD1 mRNA levels in normal and tumor cell lines were compared via qRT-PCR. All GEO accession numbers are indicated at the top of the graph, and expression levels are indicated as the means ± S.D. (E) The expressions of different rectal cancer stages were obtained from UALCAN (Normal-vs-Cancer, P<0.001; Stage1-vs-Stage2, P<0.05; Stage1-vs-Stage3, P<0.05). (F) Expressions of different colon cancer stages were obtained from UALCAN (Normal-vs-Cancer P<0.001).

MTERFD1 mRNA levels were compared between human CRC tissues and paired normal or adjacent noncancerous tissues using genomic transcriptome data downloaded from NCBI GEO. The MTERFD1 expression levels in the CRC tissues were significantly higher than those in the normal colorectal tissues (dataset accession: GSE32323 and GSE8671; Fig. 1A, 1B; P<0.0001 for both) but did not differ among CRC stages (Fig. 1E, 1F). MTERFD1 expression during inflammation-driven colorectal carcinogenesis in an AOM/DSS-treated mouse model (dataset accession: GSE31106) was also analyzed. The MTERFD1 mRNA increased after chemical induction and reached the highest level in the inflammatory colorectal mucosa at the early stage, then decreased gradually throughout the progression from dysplasia to CRC. The mean total MTERFD1 mRNA in the induced adenocarcinoma remained higher than that in the normal colorectal mucosa. No significant differences were observed between the samples at each phase and the normal mucosa, likely because there were fewer samples (n=3) at each phase. However, MTERFD1 mRNA levels in the samples with high-grade dysplasia and adenocarcinoma were significantly higher than those in the normal colorectal mucosal cells (P=0.0164; Fig. 1C). Moreover, higher MTERFD1 mRNA levels were detected in several CRC cell lines compared with those in the normal colorectal tissue and cell lines, although MTERFD1 levels varied dramatically among the CRC cell lines (Fig. 1D).

### MTERFD1 promoted proliferation and suppressed apoptosis in the CRC cell lines

**Figure 2:**
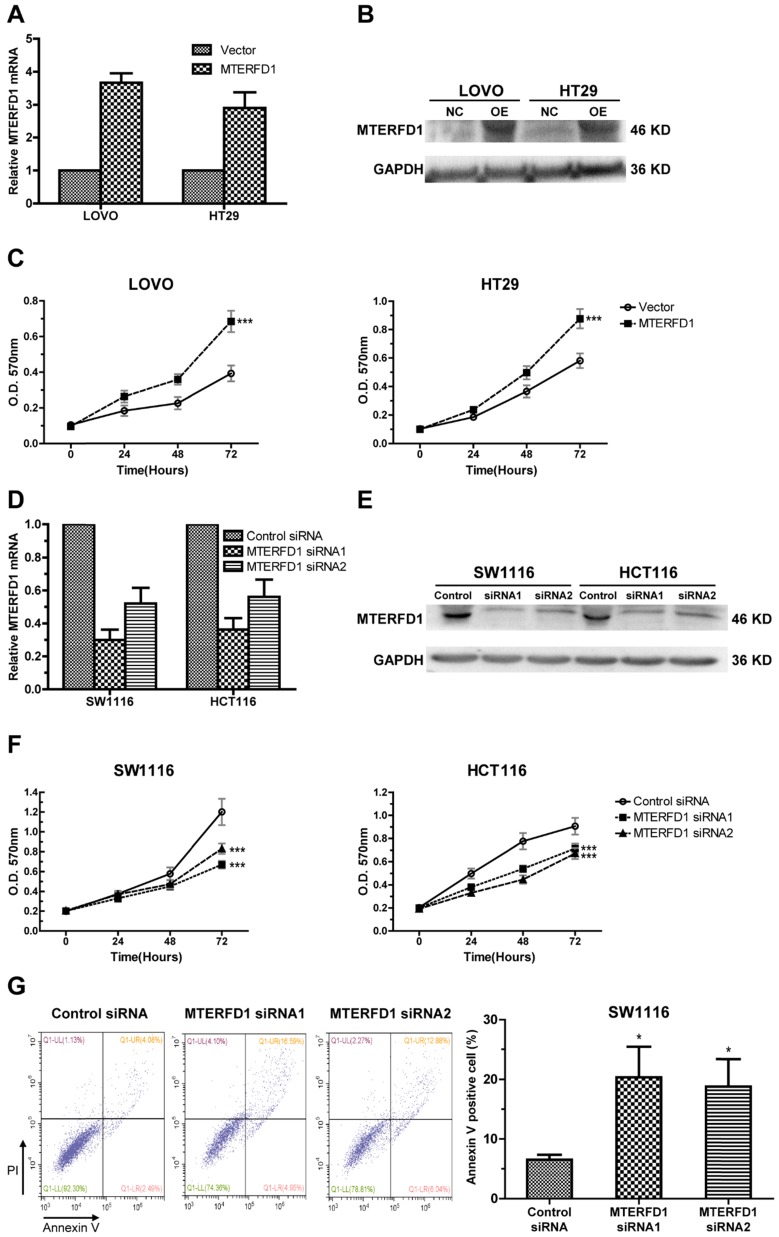
** Effects of MTERFD1 on cell proliferation and apoptosis *in vitro*.** (A, B) MTERFD1 mRNA and proteins in HT29 and LOVO cells transfected with MTERFD1-OE plasmid and control vector were evaluated via qRT-PCR (A) and western blot (B) at 72 hours post-transfection. (C) Cell growth of MTERFD1-OE LOVO and HT29 cells was monitored through MTT assays over 3 consecutive days and compared with control vector-transfected cells. (D, E) MTERFD1 mRNA and proteins in SW1116 and HCT116 cells transfected with MTERFD1 siRNA1, siRNA2 and control siRNA were evaluated via qRT-PCR (D) and western blot (E) at 72 hours post-transfection. (F) SW1116 and HCT116 cell growth was monitored via MTT assays for 3 consecutive days after siRNA transfection. (G) SW1116 cell apoptosis after transfection with MTERFD1 siRNA1, siRNA2 and control siRNA was analyzed with Annexin V and PI double-staining via flow cytometry. All experiments were performed in triplicate and a representative result is shown. Data are shown as the means ± S.D. *: P<0.05, **: P<0.01, ***: P<0.001.

To investigate the role of MTERFD1 in CRC, the MTERFD1-OE plasmid was stably transfected into LOVO and HT29 cells, which had relatively lower MTERFD1 levels among the CRC cell lines (Fig. 1D). Western blot and qRT-PCR revealed higher MTERFD1 mRNA and protein levels in the MTERFD1-OE LOVO and HT29 cells compared with those of the vector-transfected control cells (Fig. 2A, 2B). MTERFD1 overexpression induced significantly increased cell viability in LOVO and HT29 cells on the MTT assays (Fig. 2C). These results indicated that MTERFD1 promoted CRC cell proliferation. In contrast, MTERFD1 mRNA and protein were knocked down using two MTERFD1 siRNAs targeting different sequences in HCT116 and SW1116 cells (Fig. 2D, 2E), which had highest MTERFD1 levels among the CRC cell lines (Fig. 1D). Proliferation of cells transfected with two MTERFD1 siRNAs was suppressed compared with that of cells transfected with the control siRNA (Fig. 2F). Furthermore, apoptosis was significantly induced in MTERFD1-KD cells (Fig. 2G). These results indicated that MTERFD1 had a proliferative and oncogenic effect in the CRC cell lines.

### IL-6 and IL-11 were effector molecules of MTERFD1 in CRC cells

To explore the downstream effector molecules of MTERFD1, coexpression of genes with MTERFD1 in the CRC samples were analyzed using transcription profiles from the public database. IL-6 and IL-11 were significantly positively correlated with the MTERFD1 levels in the CRC samples.

**Figure 3:**
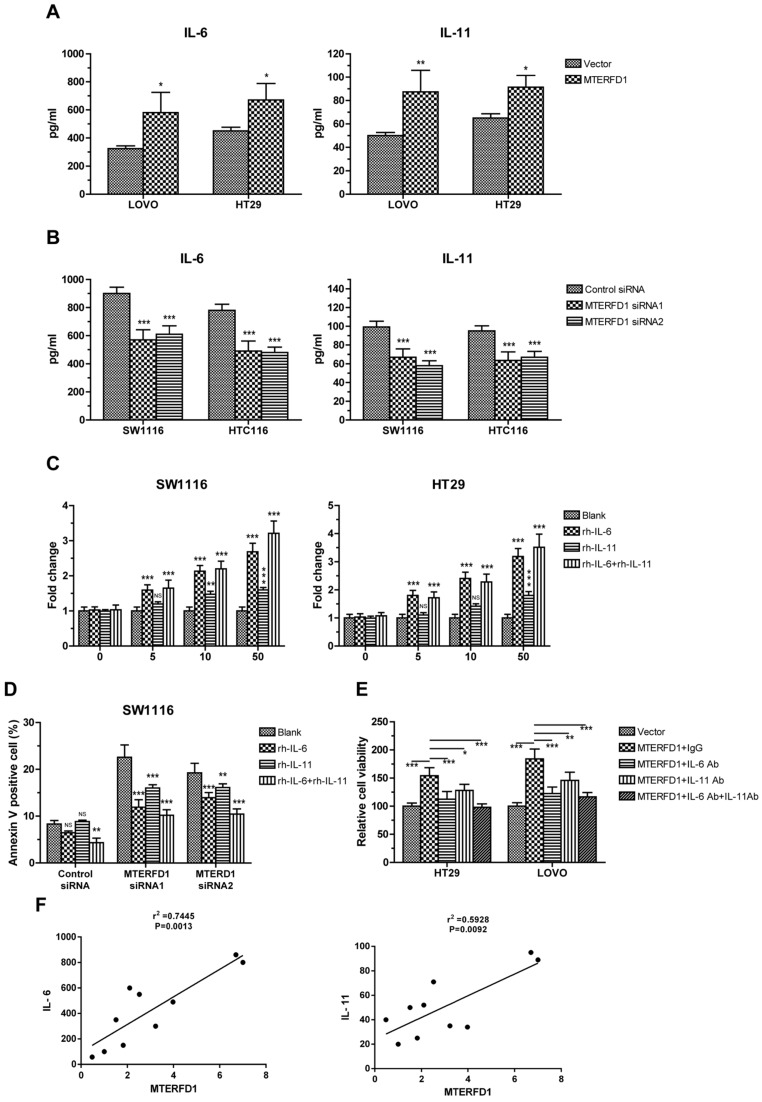
** IL-6 and IL-11 are effector molecules of MTERFD1.** (A, B) Effect of MTERFD1 alteration on IL-6 and IL-11 levels. IL-6 and IL-11 levels in the cell supernatants were analyzed by ELISA. LOVO and HT29 were transfected with either MTERFD1-OE plasmid or control vector (A). SW1116 and HCT116 were transfected with MTERFD1 siRNA1, siRNA2 and control siRNA (B). (C) SW1116 and HT29 cell growth was analyzed via MTT assays by adding IL-6 and IL-11 individually and together in cell cultures at final concentrations of 0, 5, 10, and 50 µg/L. Fold changes in cell growth were compared with cells in normal culture medium (blank). (D) SW1116 cells were transfected with MTERFD1 siRNA1, siRNA2 and control siRNA. Twenty-four hours post-transfection, the cells were cultured by adding IL-6 and IL-11 individually and together at a final concentration of 50 µg/L. Apoptosis was analyzed via flow cytometry. (E) Cells of HT29 and LOVO were transfected with either MTERFD1-OE plasmid or control vector, followed by adding nonrelevant IgG, IL-6 neutralizing antibody, IL-11 neutralizing antibody or a combination of IL-6 and IL-11 neutralizing antibodies at a final concentration of 50 µg/L. Relative cell viability was assayed via MTT assays using cells transfected with the control vector as controls. (F) Relationship between the expression level of IL6 and IL-11 and the expression level of MTERFD1 in different cell lines were compared by qRT-PCR. All experiments were performed in triplicate, and a representative results shown. Data are shown as the means ± S.D. *: p<0.05, **: p<0.01, ***: p<0.001.

To verify the correlation between IL-6 and IL-11 and MTERFD1, the IL-6 and IL-11 protein levels in the culture supernatants were assayed via ELISA in the MTERFD1-OE and MTERFD1-KD cells. Overexpression of MTERFD1 in the HT29 and LOVO cells stimulated IL-6 and IL-11 expression and secretion (Fig. 3A), while knockdown of MTERFD1 in HCT116 and SW1116 cells significantly decreased the IL-6 and IL-11 levels (Fig. 3B). MTERFD1 and IL-6/IL-11 expression were positively correlated (Fig. 3F). Adding escalating recombinant IL-6 and IL-11 proteins into the cell cultures validated the role of these proteins in CRC cell proliferation. IL-6 and IL-11 both individually and in combination enhanced cell proliferation dose-dependently compared with that of untreated cells. IL-6 was more potent in cell proliferation than was IL-11 but was less potent than IL-6 and IL-11 combined (Fig. 3C). Rescue experiments were performed to illustrate the direct roles of IL-6 and IL-11 in MTERFD1 oncogenic functions. Recombinant IL-6 and IL-11 individually attenuated SW1116 cell apoptosis in which the MTERFD1 mRNA was knocked down. Adding both cytokines exhibited partial synergistic effects on inhibiting apoptosis (Fig. 3D). When specific neutralizing antibodies were used to block IL-6 and IL-11 in the cell cultures, MTERFD1-OE-promoted cell proliferation was suppressed dramatically compared with that in cells treated with nonrelevant antibodies as controls. Adding both antibodies more strongly suppressed proliferation than did each antibody alone (Fig. 3E). These data demonstrated that IL-6 and IL-11 are the main downstream molecules of MTERFD1 functions in CRC cells.

### MTERFD1 regulated irradiation sensitivity of CRC cells in vitro

**Figure 4:**
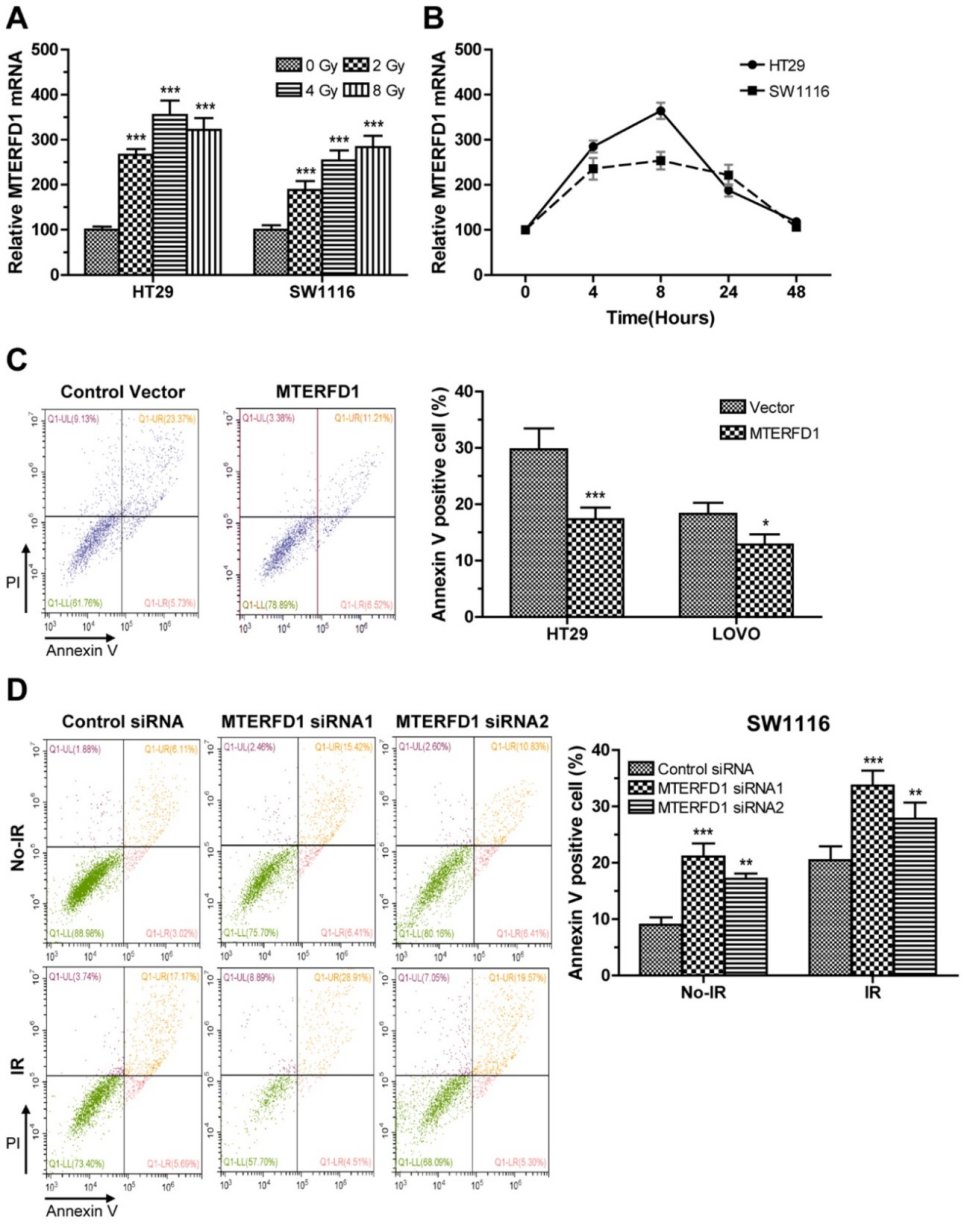
** MTERFD1 is associated with irradiation sensitivity in CRC cells.** (A, B) Effect of irradiation on MTERFD1 expression. (A) HT29 and SW1116 cells were irradiated with 0, 2, 4, or 8 Gy. The relative levels of MTERFD1 mRNA were determined via qRT-PCR using cells treated with 0 Gy as controls. (B) HT29 and SW1116 cells were irradiated with 4 Gy, and relative levels of MTERFD1 mRNA were determined via qRT-PCR at 0, 4, 8, 24 and 48 hours post-irradiation. (C, D) Effect of MTERFD1 expression on irradiation sensitivity. (C) HT29 and LOVO cells transfected with either MTERFD1-OE plasmid or control vector were irradiated with 4 Gy. Apoptosis was analyzed via flow cytometry 24 hours post-irradiation. (D) SW1116 cells transfected with MTERFD1 siRNA1, siRNA2 and control siRNA were irradiated with 4 Gy. Apoptosis of cells both with and without irradiation was analyzed via flow cytometry 24 hours post-irradiation. All experiments were performed in triplicate and a representative result is shown. Data are shown as the means ± S.D. *: p<0.05, **: p<0.01, ***: p<0.001.

Alterations in MTERFD1 expression in the irradiated CRC cells were analyzed via qRT-PCR. MTERFD1 mRNA levels in HT29 and SW1116 cells exposed to 2, 4 and 8 Gy irradiation were significantly increased compared with those in the nonirradiated cells (Fig. 4A). In time courses of MTERFD1 alteration after 4 Gy of irradiation in both cell lines, the mRNA levels increased and peaked at 8 hours post-irradiation, then returned to pre-irradiation levels at 48 hours post-irradiation (Fig. 4B). To investigate the role of MTERFD1 on irradiation sensitivity of CRC cells, the CRC cell lines with MTERFD1-OE or MTERFD1-KD were exposed to 4 Gy of irradiation and tested for apoptosis. MTERFD1-OE significantly mitigated irradiation-induced apoptosis in HT29 (P<0.001) and LOVO (P<0.05; Fig. 4C) cells. In contrast, MTERFD1-KD with two siRNAs in SW1116 cells significantly enhanced irradiation-induced apoptosis (Fig. 4D; siRNA1 P<0.001, siRNA2 P<0.01). These results indicated that MTERFD1 regulated irradiation-induced apoptosis or cell death *in vitro*, and higher MTERFD1 levels contributed to irradiation resistance in CRC.

### IL-6 and IL-11 were involved in MTERFD1-regulated irradiation sensitivity in CRC cells

**Figure 5:**
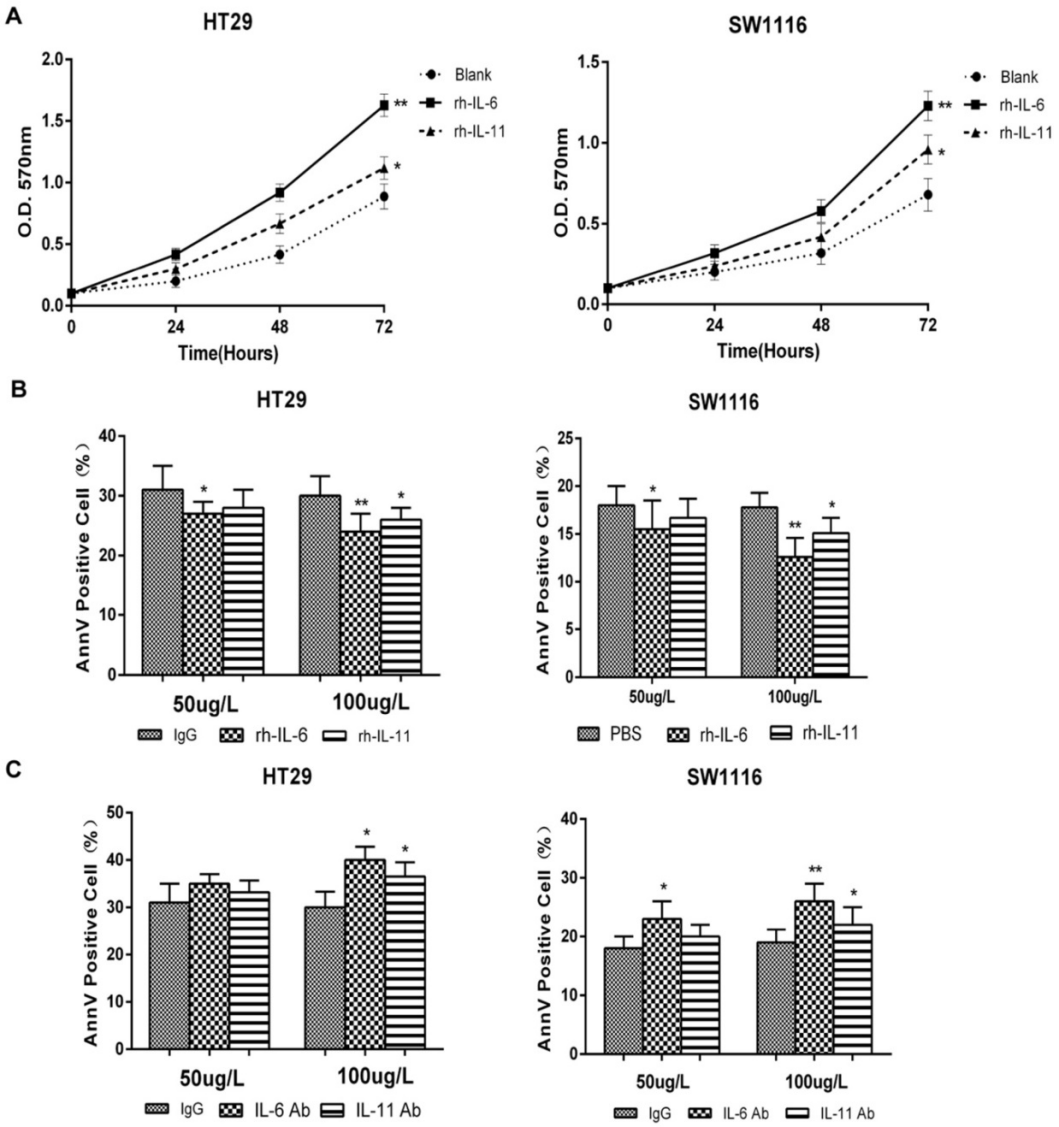
** IL-6 and IL-11 are associated with irradiation sensitivity in CRC cells.** (A) SW1116 and HT29 cell growth was monitored via MTT assays for 3 consecutive days after administering rh-IL-6 and rh-IL-11. (B) HT29 and SW1116 cells were irradiated with 4 Gy, then incubated with recombinant IL-6 and IL-11 (50 µg/L and 100 µg/L) in culture medium for 3 days. Apoptosis was analyzed using flow cytometry. (C) HT29 and SW1116 cells were irradiated with 4 Gy, then incubated with nonrelevant IgG and neutralizing antibodies of IL-6 and IL-11 (50 µg/L and 100 µg/L) in culture medium for 3 days. Apoptosis was analyzed using flow cytometry. All experiments were performed in triplicate and a representative result is shown. Data are shown as the means ± S.D. *: p<0.05, **: p<0.01.

**Figure 6:**
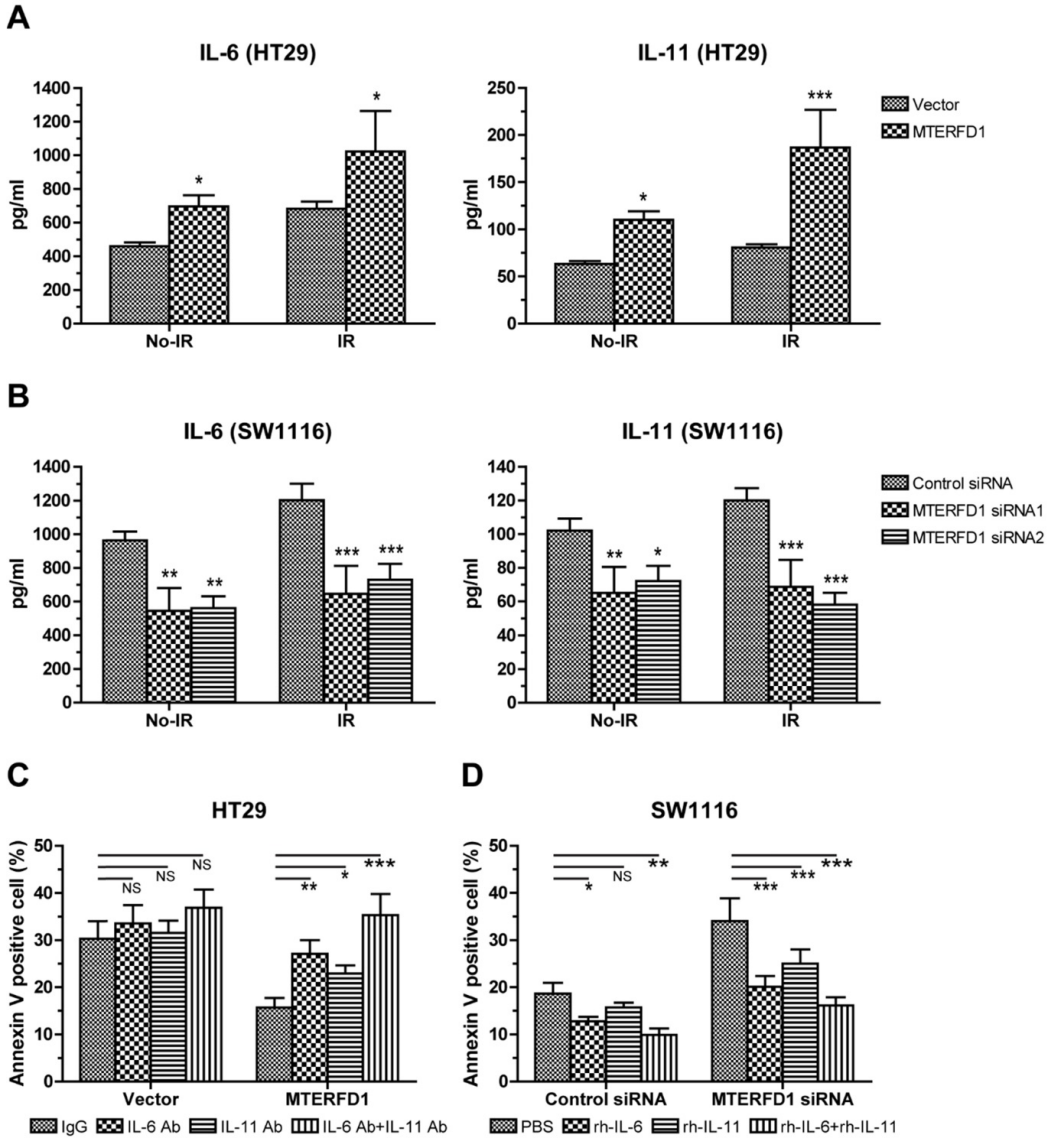
** IL-6 and IL-11 are effect molecules of MTERFD1 on irradiation sensitivity.** (A) HT29 cells were transfected with either MTERFD1-OE plasmid or control vector, followed by irradiation with 4 Gy. IL-6 and IL-11 were analyzed by ELISA in the supernatants of cells treated with and without irradiation. (B) SW1116 cells were transfected with MTERFD1 siRNA1, siRNA2 and control siRNA, then irradiated with 4 Gy. IL-6 and IL-11 were analyzed using ELISA on the supernatants of irradiated and nonirradiated cells. (C) MTERFD1-OE and control vector-transfected HT29 cells were irradiated with 4 Gy, then incubated with nonrelevant IgG and neutralizing antibodies of IL-6 and IL-11 in culture medium for 3 days. Apoptosis was analyzed via flow cytometry. (D) The MTERFD1-KD and control siRNA-transfected SW1116 cells were irradiated with 4 Gy, then incubated with recombinant IL-6 and IL-11 in culture medium for 3 days. Apoptosis was analyzed via flow cytometry. All experiments were performed in triplicate, and a representative result is shown. Data are shown as the means ± S.D. *: p<0.05, **: p<0.01, ***: p<0.001.

After identifying the regulatory role of MTERFD1 on the irradiation sensitivity of CRC cells, we determined whether IL-6 and IL-11 also played key roles in this process. MTERFD1-OE in CRC cells upregulated IL-6 and IL-11 expression (Fig. 3A), and IL-6 and IL-11 promoted HT29 and SW1116 cell proliferation (Fig. 5A). Adding different concentrations of rh-IL-6 and rh-IL-11 to HT29 and SW1116 cells under 4 Gy irradiation inhibited CRC cell apoptosis, and increasing the dose strengthened the inhibitory effect (Fig. 5B). Similarly, adding different concentrations of IL-6Ab and IL-11Ab to HT29 and SW1116 cell lines promoted apoptosis of CRC cells under irradiation (Fig. 5C). MTERFD1-OE upregulated IL-6 and IL-11 expression in the HT29 cells without irradiation compared with cells transfected with the control vector (Fig. 6A). Furthermore, irradiation of the MTERFD1-OE HT29 cells led to higher IL-6 and IL-11 levels in both the control and vector-transfected cells with irradiation and MTERFD1-OE HT29 cells without irradiation (Fig. 6A). MTERFD1 knockdown by siRNAs significantly decreased the IL-6 and IL-11 levels in SW1116 cells treated with and without irradiation (Fig. 6B). To confirm the role of IL-6 and IL-11 on MTERFD1 regulation of irradiation sensitivity, rescue experiments were performed in CRC cells under irradiation using neutralizing antibodies and recombinant IL-6 and IL-11. The specific neutralizing antibodies against IL-6 and IL-11 when used individually or in combination impaired the role of MTERFD1-OE on attenuating irradiation-induced apoptosis in HT29 cells (Fig. 6C). The neutralizing antibodies did not significantly increase the irradiation-induced apoptosis in control vector-transfected HT29 cells (Fig. 6C). Conversely, using IL-6 and IL-11 both individually and in combination alleviated apoptosis in the irradiated MTERFD1-KD SW1116 cells, while IL-6 and combined IL-6 and IL-11 also alleviated apoptosis in the irradiated control siRNA-transfected SW1116 cells (Fig. 6D). These results indicated that IL-6 and IL-11 are the key downstream effectors of MTERFD1 regulation on irradiation sensitivity of CRC.

## Discussion

Although regulation of mitochondrial genes by MTERFD1 has been thoroughly investigated 8, 9 , the role of MTERFD1 in tumorigenesis has been unreported until recently 10. MTERFD1's role in CRC remains uncertain. In this study, the correlation between high MTERFD1 expression and CRC tumorigenesis was first identified by comparing clinical CRC tissues to paired adjacent normal tissues by analyzing genomic transcription data from NCBI GEO. Second, MTERFD1 expression was verified to be higher in CRC cell lines than in normal colorectal cell lines at the mRNA level. Third, the oncogenic role of MTERFD1 on CRC was demonstrated *in vitro* by promoting cell proliferation with MTERFD1-OE and inducing apoptosis with MTERFD1-KD. Intriguingly, in the mouse model of chemically induced inflammation-driven CRC, the MTERFD1 mRNA levels peaked in the inflammatory colorectal mucosa in the early stage, then decreased gradually throughout the progression to CRC. MTERFD1 mRNA levels in induced dysplasia and CRC tissues remained significantly higher than that in normal mucosa, implying that MTERFD1 may also be involved in the inflammatory processes in the early stages of the CRC onset.

In normal gastrointestinal tract tissue, IL-6 is a key cytokine in controlling tissue homeostasis and barrier function. However, under pathological conditions, IL-6 is also crucial to maintain chronic inflammation, which is closely related to initiation and progression of CRC-associated inflammation 14, 15. The JAK/STAT3 signaling pathway, activated by IL-6, is involved in CRC development by facilitating proliferation and inhibiting apoptosis and other protumorigenic pathways^16^, ^17^. IL-11, a cytokine of the IL-6 family, was recently reported to be more prominent than IL-6 in the progression of inflammation-associated gastrointestinal cancer. IL-11 activity in CRC is also strongly correlated with the STAT3 pathway 18.

In addition, upregulating IL-6 (and IL-11) in normal and malignant colorectal tissues induces activation of NF-κB, a key transcription factor regulating immunological and inflammatory responses. NF-κB and its pathway participate in inflammation and inflammation-associated tumorigenesis and metastasis in inflammatory bowel disease (IBD) and colitis-associated cancer (CAC)^19^. Moreover, NF-κB interacts physically with STAT3 to facilitate NF-κB binding to target promoters. STAT3 mediates NF-κB acetylation by recruiting the acetyltransferase p300 to prolong NF-κB nuclear retention^16^, which establishes the crosstalk between the NF-κB and STAT3 signaling pathways. NF-κB and STAT3 cooperatively regulate several cytokines and chemokines including IL-6 16, which alone activates STAT3 and generates an IL-6/STAT3/NF-κB positive-feedback circuit, which plays a role in chronic colorectal inflammation and cancer. IL-11 expression was reduced in NF-κB signaling-defective myeloid cells in carcinogen-induced CRC 20.

The effectors or downstream targets of MTERFD1 as an oncogene remain unknown. Because IL-6 and IL-11 have important effects on gastrointestinal chronic inflammation as well as onset and progression of inflammation-associated CRC, we hypothesized that both IL-6 and IL-11 might be associated with MTERFD1 function in CRC development. We demonstrated that upregulation and downregulation of MTERFD1 were positively correlated with IL-6 and IL-11 expression and secretion in cultured CRC cells. Furthermore, adding recombinant cytokines to the culture medium abolished apoptosis due to MTERFD1 downregulation, while neutralizing cytokines by antibodies inhibited cell proliferation due to MTERFD1 upregulation. Our results indicated that MTERFD1 influenced CRC development by at least partly regulating CRC cell-derived expression of IL-6 and IL-11. In the *in vitro* cell cultures, IL-6 and IL-11 were produced and secreted from CRC cells and had an autocrine effect on the CRC cells. The autocrine IL-6 signaling loop is reported to promote progression of multiple epithelial cancers, such as CAC^16^, lung adenocarcinoma^22^, and breast cancer^23^-^25^, by excessive activation of STAT3 and NF-kB 21. Autocrine IL-11 also mediates tumorigenicity in hypoxic human cancer cells by activating STAT activation^26^. To our knowledge, this work is the first to report that MTERFD1 is a novel molecule that regulates the IL-6 and IL-11 autocrine signaling loops in CRC cells for its oncogenic functions. However, in human CRC tissues and CRC mouse models, IL-6 is produced mostly from activated myeloid cells in the tumor microenvironment, while IL-11 is produced mostly by cancer-associated fibroblasts (CAFs) and myeloid cells. Our results did not exclude the possibility that MTERFD1 might induce IL-6 and IL-11 production in these tumor stromal cells and infiltrated immune cells to promote CRC development in a paracrine manner; this possibility requires further investigation. Because activation of the NF-κB and STAT pathways is frequently associated with IL-6 and IL-11 signaling in CRC, both pathways may be engaged in MTERFD1's oncogene functions, which requires further investigation.

**Figure 7:**
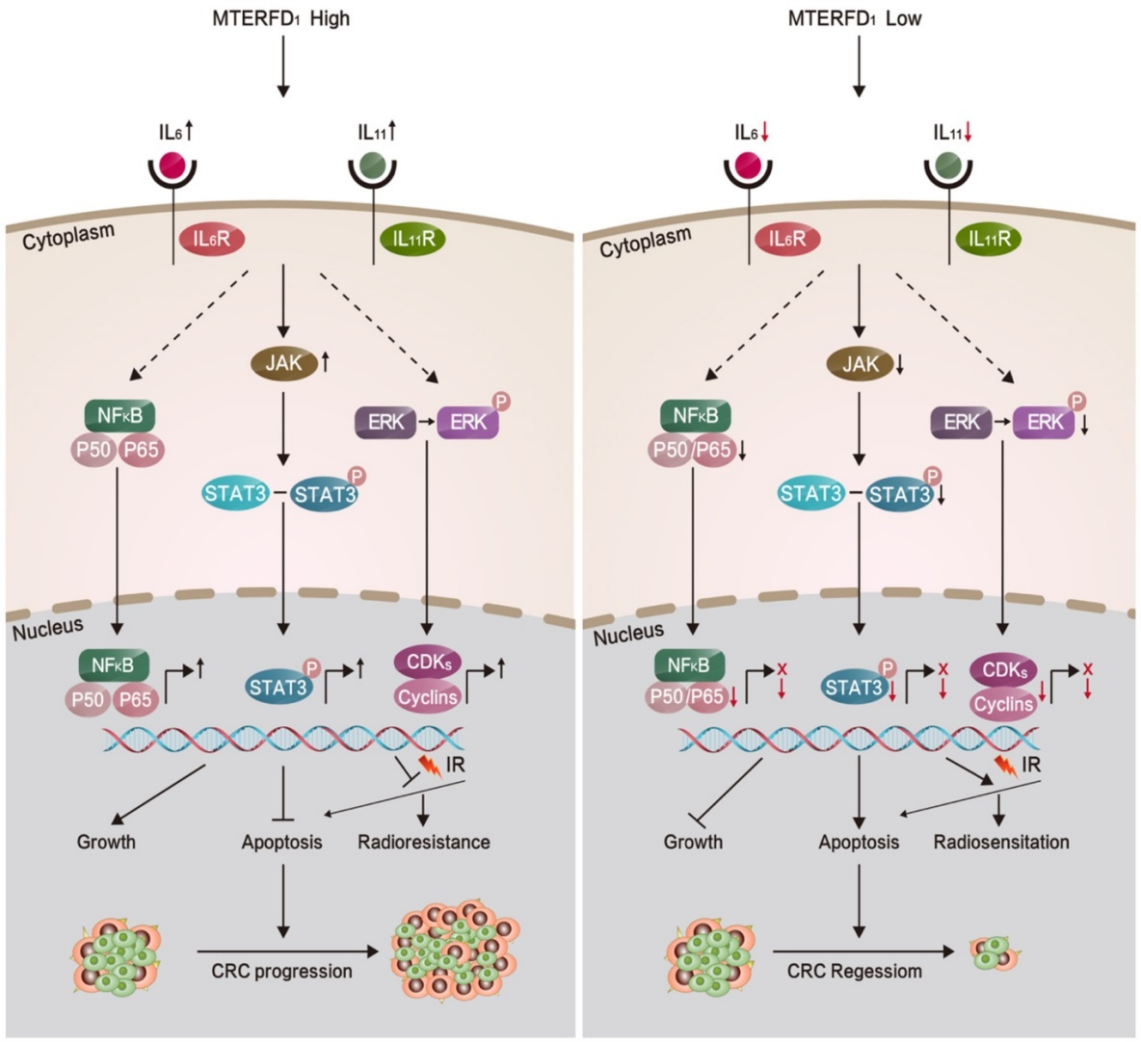
Proposed model illustrating the roles of MTERFD1 during CRC carcinogenesis and radiosensitivity.

Because MTERFD1 is an oncogene in CRC development, we investigated whether MTERFD1 plays a direct role in mediating irradiation resistance in CRC cells. Our results showed that irradiation of CRC cells upregulated MTERFD1 expression. Upregulating MTERFD1 in CRC cells by transfecting the OE plasmid reduced irradiation-induced apoptosis, resulting in increased irradiation resistance *in vitro*. Conversely, downregulating MTERFD1 expression by siRNAs enhanced irradiation-induced apoptosis, indicating increased radiosensitivity. Thus, MTERFD1 expression in the CRC cells was directly correlated with *in vitro* cell response to irradiation treatment. Because IL-6 and IL-11 play catalytic roles in colorectal cancer^27^-^29^ and many other tumors^18^, ^30^-^32^, and IL-1^33^, IL-11β^34^, IL-2^35^ IL-4^36^, IL-6^35^-^37^, and IL-11^38^ have been confirmed to be resistant to radiotherapy, we identified that alterations in IL-6 and IL-11 expression were involved in the irradiation resistance of CRC cells *in vitro* as the downstream effectors of MTERFD1. Fig. 7 summarizes this process in a schematic diagram. This is consistent with previous reports that IL-6 and IL-11 expression and activated signaling pathways were positively linked with irradiation resistance in lung cancer, liver tumors, prostate cancer and leukemia^39^-^43^. Our results suggest that MTERFD1 is a radioresistance factor and could be a potential prognostic and therapeutic marker for radiotherapy for CRC. Neutralizing antibodies (Abs) against IL-6 and IL-11 with MTERFD1-OE restored the radiosensitivity in the CRC cells. Specific Abs against IL-6 and IL-11, as well as inhibitors of downstream signaling, especially NF-κB and STAT signals, have been used to treat inflammatory, autoimmune and malignant diseases^44^, ^45^. This suggests that as downstream effectors of MTERFD1 in radioresistance, these Abs and inhibitors may abrogate MTERFD1-induced radioresistance. Additional studies should validate the roles of these Abs and inhibitors in improving irradiation response during radiotherapy for colorectal cancer.
